# Association of basal metabolic rate and fuel oxidation in basal conditions and during exercise, with plasma S-klotho: the FIT-AGEING study

**DOI:** 10.18632/aging.102100

**Published:** 2019-08-07

**Authors:** Francisco J. Amaro-Gahete, Alejandro De-la-O, Lucas Jurado-Fasoli, Jonatan R. Ruiz, Manuel J. Castillo

**Affiliations:** 1 EFFECTS-262 Research Group, Department of Medical Physiology, School of Medicine, University of Granada, Granada, Spain; 2 PROmoting FITness and Health through Physical Activity Research Group (PROFITH), Sport and Health University Research Institute (iMUDS), Department of Physical Education and Sports, Faculty of Sport Sciences, University of Granada, Granada, Spain

**Keywords:** aging, MFO, basal fat oxidation, basal metabolic rate, Fat _max_

## Abstract

S-klotho, the shed form of α-klotho, is thought to be an ageing suppressor with functions related to the physiology of energy metabolism. However, it remains unknown whether ageing biomarkers such as S-klotho and/or chronological ageing are associated in any way with basal metabolic rate (BMR) and fuel oxidation in basal conditions and during exercise. The present work investigates the association of BMR and fuel oxidation in basal conditions and during exercise, with plasma S-klotho in middle-aged, sedentary adults. BMR was measured by indirect calorimetry in 74 such subjects (53% women; age 53.7±5.1 years) following standard procedures, and their fuel oxidation estimated via stoichiometric equations. The maximal fat oxidation during exercise (MFO) and the intensity of exercise that elicits MFO (Fat_max_) were determined using a walking graded exercise test. No relationship was seen between BMR and plasma S-klotho (P>0.1), although both basal fat oxidation and MFO showed positive associations with this protein (both P<0.001); these relationships persisted after controlling for age, sex and fat mass. However, no significant associations were seen between BMR, basal fat oxidation or MFO and chronological age (all P>0.1). The present findings suggest that basal fat oxidation and MFO are strongly associated with plasma S-klotho in middle-aged sedentary adults. These results support the idea that metabolic flexibility is a powerful predictor of biological ageing.

## Introduction

Life expectancy in Europe has generally increased in recent decades. In 2012, 17% of the European Union population was aged 65 years or older, a percentage expected to rise to 25% by 2035, and to 30% in 2060 [1]. However, a longer life expectancy does not necessarily mean healthy ageing; it can mean extra years of suffering chronic disease, particularly metabolic illnesses such us obesity and diabetes mellitus type II [1,2].

Ageing is characterized by a progressive decline in one's metabolic and physiological functions [3], the associated dysregulation of nutrient sensitivity, mitochondrial dysfunction and cellular apoptosis eventually becoming harmful [4] Ageing is associated with a progressive decline in the basal metabolic rate (BMR), meal-induced thermogenesis and physical activity [5], resulting in a reduced total energy expenditure. In part, this is responsible for the gradual weight increase and the deposition of visceral adipose tissue seen during ageing, which places people at greater risk of cardiometabolic disease and all-cause mortality [6].

Over the last decade, numerous studies have examined the association between basal fuel oxidation and ageing-related diseases, and a potential role for this oxidation has been proposed in the pathogenesis of subclinical atherosclerosis, hypertriglyceridaemia, liver steatosis and ventricular cardiac remodelling [7―9]. Ageing is positively associated with visceral adiposity, but in a study involving a large and heterogeneous adult population, no relationship was observed between basal substrate oxidation and chronological age [3]. Recent studies have suggested that chronological age is but a crude indicator of ageing. Specific ageing biomarkers provide a more accurate picture; indeed, they provide a reliable tool for understanding and assessing ageing [10].

The α-klotho gene is thought to suppress ageing, extending life expectancy when it is overexpressed and inducing premature ageing when it is defective [11,12]. It is mainly expressed in the kidney, the parathyroid glands and the brain; its product is a type-1 single-pass transmembrane glycoprotein, the ectodomain of which is shed and released into the systemic circulation in soluble form (S-klotho) [13]. S-klotho has several functions related to the physiology of energy metabolism [14], including the regulation of glucose uptake, the enhancement of insulin sensitivity, the attenuation of cellular oxidative stress, and the suppression of chronic inflammation [15―17], which together are thought to invest it with anti-ageing properties. A recent study showed that plasma S-klotho is lower in individuals with diabetes mellitus type II, and therefore a potential biomarker of this disease [18]. It thus seems plausible that individuals with a reduced BMR and an altered fuel oxidation in basal conditions and during exercise may have lower plasma S-klotho. The literature contains no studies on how BMR and fuel oxidation in basal conditions and during exercise may be related to chronological ageing, or whether they have any relationship with ageing biomarkers such as S-klotho. The aim of the present work was to investigate the relationship of BMR and fuel oxidation in basal conditions and during exercise, with plasma S-klotho.

## RESULTS

**Figure 1:**
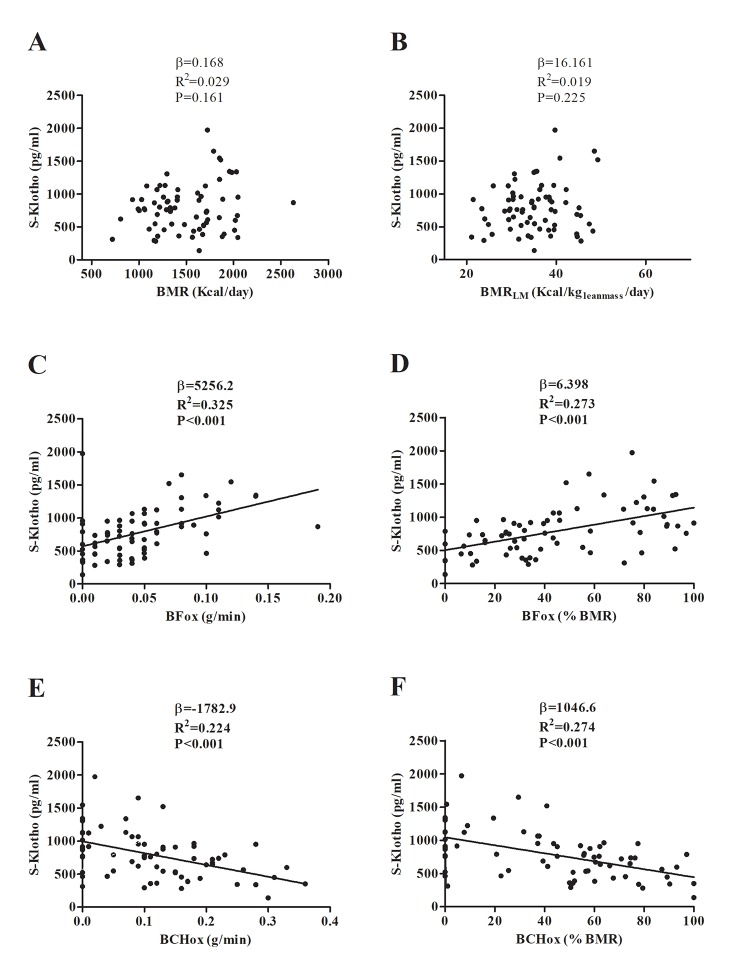
Association between basal metabolic rate (BMR) (**A, B**), basal fat oxidation (BFox) (**C, D**) and basal carbohydrate oxidation (BCHox) (**E, F**) with plasma S-klotho levels. β (unstandardized regression coefficient), R^2^, and P are from simple linear regression analysis. Abbreviations: BMR_LM_; Basal Metabolic Rate relative to lean mass.

Table 1 summarises the descriptive characteristics of the study subjects. BMR and BMR_LM_ showed no significant association with plasma S-klotho (Figure 1A and 1B; P>0.1), a result that persisted after controlling for age, sex and percentage fat mass (Table 2; P>0.05). A significant, negative association was detected between BCHox (expressed in g/min, and in % BMR) and plasma S-klotho (1E and 1F; all P≤0.001), while a significant positive association was seen between BFox (expressed in g/min, and in % BMR) and plasma S-klotho (Figure 1C, and 1D; all P<0.001). These associations persisted after controlling for age, sex, and percentage fat mass (Table 2; P≤0.01).

**Table 1:** Study participant characteristics.

	**N**	**All**		**N**	**Men**		**N**	**Women**	
Age (years)	74	53.7	(5.1)	35	54.4	(5.3)	39	53.0	(5.0)
S-Klotho plasma levels (pg/ml)	73	775.3	(363.7)	34	814.1	(452.2)	39	741.4	(265.6)
**Anthropometry and body composition**									
Weight (kg)	74	75.7	(15.0)	35	87.4	(11.0)	39	65.3	(9.3)*
Height (cm)	74	167.8	(9.8)	35	175.8	(6.5)	39	160.7	(6.1)*
Body mass index (kg/m^2^)	74	26.7	(3.8)	35	28.3	(3.6)	39	25.3	(3.3)*
Fat mass (kg)	74	30.0	(8.4)	35	30.9	(9.8)	39	29.2	(7.1)
Fat mass (%)	74	39.9	(9.1)	35	34.7	(8.0)	39	44.5	(7.4)*
Fat mass index (kg/m^2^)	74	10.7	(3.1)	35	10.0	(3.2)	39	11.4	(2.9)
Visceral adipose tissue (g)	74	789.7	(387.1)	35	972.4	(392.0)	39	625.8	(303.4)*
Lean mass (kg)	74	43.5	(11.7)	35	53.9	(6.5)	39	34.1	(5.8)*
Lean mass index (kg/m^2^)	74	15.2	(2.9)	35	17.5	(20.0)	39	13.2	(1.8)*
**Basal metabolic rate and fuel oxidation under post-fast baseline conditions**									
BMR (kcal/day)	71	1508.4	(364.5)	34	1805.5	(244.8)	37	1235.5	(208.4)*
BMR_LM_ (kcal/kg_leanmass_/day)	71	35.2	(7.2)	34	33.6	(5.3)	37	36.7	(8.4)
BFox (g/min)	71	0.053	(0.040)	34	0.064	(0.050)	37	0.042	(0.025)*
BFox (% BMR)	71	45.6	(30.0)	34	45.6	(32.7)	37	45.6	(27.7)
BCHox (g/min)	71	0.112	(0.096)	34	0.138	(0.115)	37	0.089	(0.069)*
BCHox (% BMR)	71	41.8	(32.0)	34	44.0	(34.7)	37	39.8	(29.0)
**Fuel oxidation during exercise**									
MFO (g/min)	71	0.29	(0.09)	34	0.35	(0.09)	37	0.23	(0.04)*
MFO_LM_ (g/kg_leanmass_/min)	71	6.72	(1.61)	34	6.43	(1.49)	37	6.99	(1.70)
Fat_max_ (%VO_2_max)	71	43.0	(10.4)	34	41.6	(10.3)	37	44.3	(10.6)
**Cardiorespiratory fitness**									
VO_2_max (ml/min)	71	2339.2	(657.2)	34	2915.4	(373.2)	37	1809.7	(332.5)*
VO_2_max (ml/kg/min)	71	30.5	(5.6)	34	33.3	(4.5)	37	27.9	(5.3)*

Data are presented as means (standard deviation). *Significant differences between sexes obtained via the T-Student unpaired-samples test (P<0.05). Abbreviations: BMR; Basal Metabolic Rate, BMR_LM_; Basal Metabolic Rate relative to Lean Mass, BFox; Basal Fat Oxidation, BCHox; Basal Carbohydrate Oxidation, MFO; Maximal Fat Oxidation, MFO_LM_; Maximal Fat Oxidation relative to Lean Mass, Fat_max_; Intensity of exercise that elicits MFO, VO_2_max; Maximum Oxygen Uptake.

**Table 2:** Association of basal metabolic rate, basal fat oxidation, basal carbohydrate oxidation, maximal fat oxidation and the intensity of exercise that elicits maximal fat oxidation (Fat_max_) with plasma S-klotho concentration and chronological age, adjusted for age (Model 1), sex (Model 2), percentage fat mass (Model 3) and age, sex and percentage of fat mass (Model 4 / Model 4*: adjusted for sex and percentage of fat mass).

	**Plasma S-klotho concentration**								
		Model 1		Model 2		Model 3		Model 4	
		P value	β	P value	β	P value	β	P value	β
BMR (kcal/day)		0.209	0.184	0.203	0.247	0.670	0.051	0.497	0.089
BMR_LM_ (kcal/ kg_leanmass_/day)		0.150	10.495	0.091	15.868	0.490	9.180	0.329	4.611
BFox (g/min)		**<0.001**	**3340.712**	**<0.001**	**5380.689**	**<0.001**	**4701.526**	**<0.001**	**2829.975**
BFox (% BMR)		**0.001**	**3.536**	**<0.001**	**6.434**	**<0.001**	**5.895**	**0.002**	**3.211**
BCHox (g/min)		**0.010**	**-887.501**	**<0.001**	**-2038.484**	**<0.001**	**-1723.932**	**<0.001**	**-1057.784**
BCHox (% BMR)		**0.002**	**-3.204**	**<0.001**	**-6.145**	**<0.001**	**-5.580**	**0.002**	**-3.034**
MFO (g/min)		**<0.001**	**1312.915**	**0.036**	**1429.228**	**0.009**	**715.126**	**0.003**	**1298.556**
MFO_LM_ (g/kg_leanmass_/min)		0.956	1.120	0.294	-29.664	0.891	3.964	0.181	35.482
Fat_max_ (% VO_2_max)		0.724	1.108	0.133	-6.349	0.078	-6.886	0.081	31.824
	**Chronological age**								
				Model 2		Model 3		Model 4*	
				P value	β	P value	β	P value	β
BMR (kcal/day)				0.132	-0.004	0.627	0.001	0.075	-0.005
BMR_LM_ (kcal/ kg_leanmass_/day)				0.071	0.150	0.312	0.098	0.386	0.082
BFox (g/min)				0.453	-55.185	0.370	-44.989	0.418	-50.895
BFox (% BMR)				0.186	-1.426	0.505	0.042	0.106	0.118
BCHox (g/min)				0.676	-0.471	0.301	0.065	0.114	0.128
BCHox (% BMR)				0.279	-1.163	0.459	0.046	0.117	0.115
MFO (g/min)				0.326	-8.231	0.678	2.874	0.315	-8.199
MFO_LM_ (g/kg_leanmass_/min)				0.089	0.627	0.282	0.426	0.298	0.401
Fat_max_ (% VO_2_max)				0.106	-1.835	0.188	0.085	0.094	0.137

P value of multiple-regression analysis. β (unstandardized regression coefficient). Abbreviations: BMR; Basal Metabolic Rate, BMRLM; Basal Metabolic Rate relative to lean mass, BFox; Basal Fat Oxidation, BCHox; Basal Carbohydrate Oxidation, MFO; Maximal Fat Oxidation, MFOLM; Maximal Fat Oxidation relative to lean mass, Fatmax; Intensity of exercise that elicits MFO, VO2max; Maximum Oxygen Uptake.

**Figure 2:**
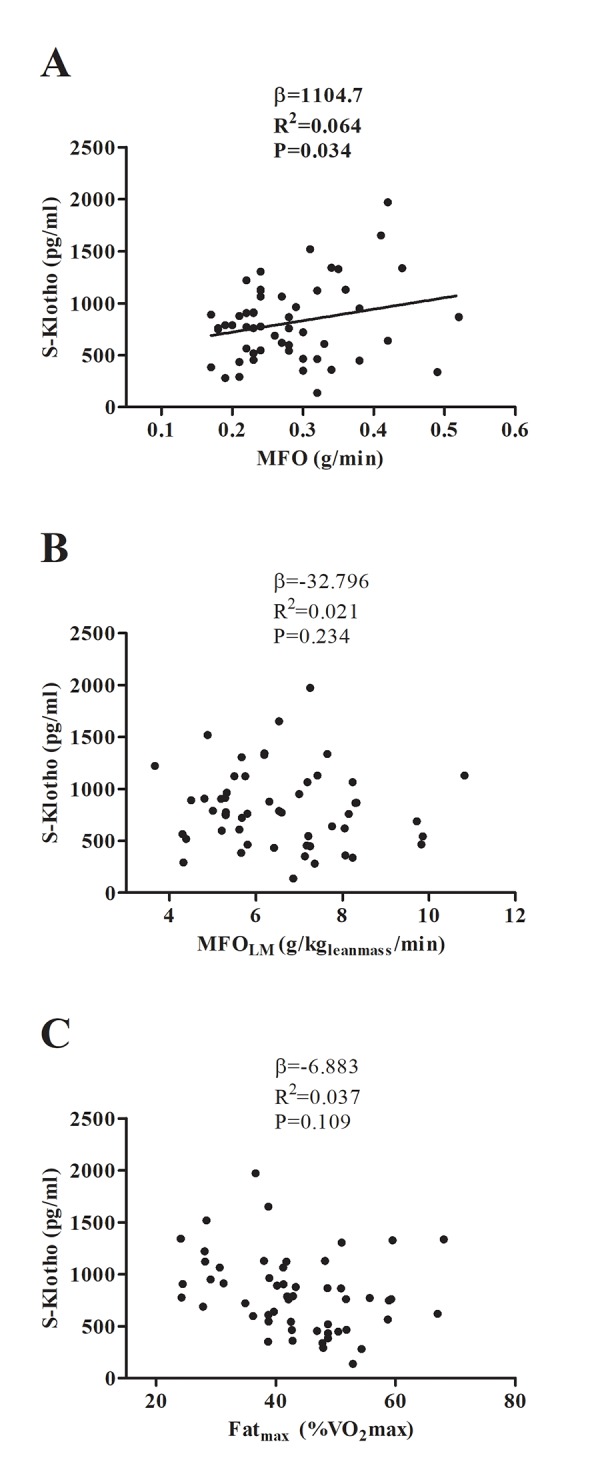
Association between maximal fat oxidation (MFO) (**A, B**), and the intensity of exercise that elicits MFO (Fat_max_) (**C**) with plasma S-klotho. β (unstandardized regression coefficient), R^2^ and P are from simple linear regression analysis. Abbreviations: MFO; Maximal Fat Oxidation, MFO_LM_; Maximal Fat Oxidation relative to lean mass, Fat_max_; Intensity of exercise that elicits MFO, VO_2_max; Maximal Oxygen Uptake.

MFO was significantly associated with plasma S-klotho (Figure 2A; P=0.034, β=1104.7) even after controlling for age, sex, and percentage fat mass (Table 2; P≤0.04). Neither MFO_LM_ nor Fat_max_ showed any relationship with plasma S-klotho (Figure 2B and 2C; all P>0.1), a finding that persisted after adjusting for age, sex, and percentage fat mass (Table 2; P>0.08).

**Figure 3:**
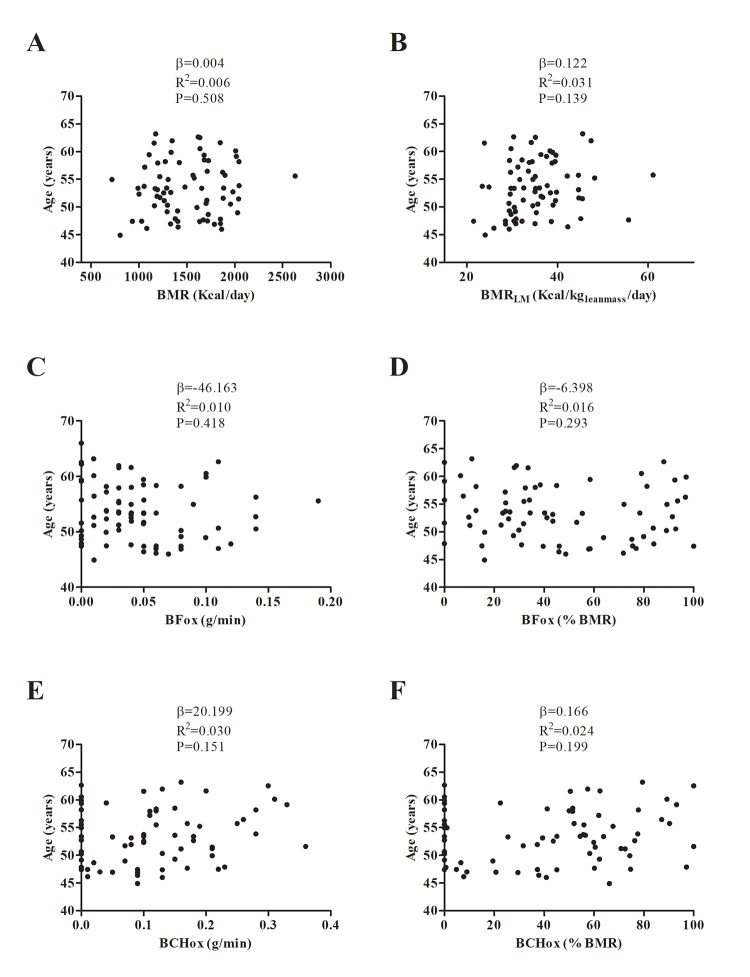
Association between basal metabolic rate (BMR) (**A, B**), basal fat oxidation (BFox) (**C, D**) and carbohydrate oxidation (BCHox) (**E, F**) with age. β (unstandardized regression coefficient), R^2^ and P are from a simple linear regression analysis. Abbreviations: BMR_LM_; Basal Metabolic Rate relative to lean mass.

**Figure 4:**
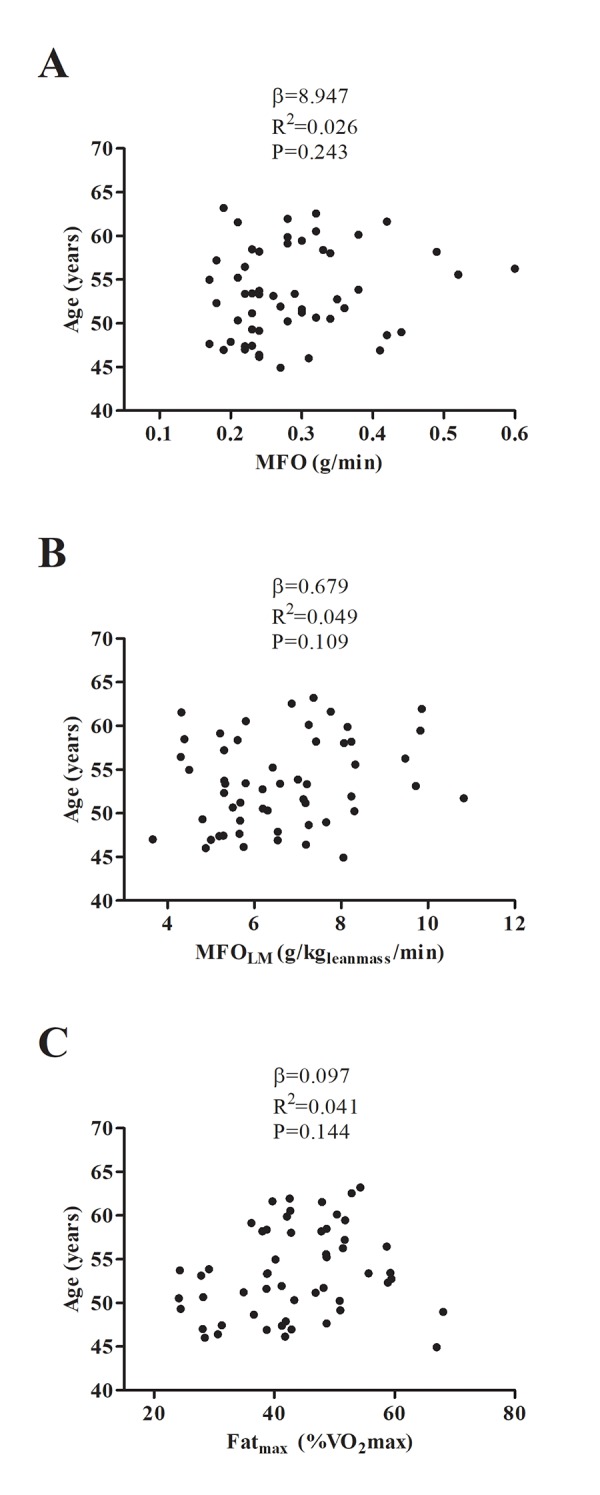
Association between maximal fat oxidation (MFO) (**A, B**), and the intensity of exercise that elicits MFO (Fat_max_) (**C**) with age. β (unstandardized regression coefficient), R^2^ and P are from a simple linear regression analysis. Abbreviations: MFO; Maximal Fat Oxidation, MFO_LM_; Maximal Fat Oxidation relative to lean mass, Fat_max_; Intensity of exercise that elicits MFO, VO_2_max; Maximal Oxygen Uptake.

Neither BMR, BMR_LM_, BFox, BCHox, MFO, MFO_LM_ nor Fat_max_ showed an association with chronological age (Figure 3 and Figure 4; all P>0.1); these findings persisted after adjusting for sex and percentage fat mass (Table 2).

All of the above-mentioned analyses were also run adjusting for visceral adipose tissue, VO_2_max, objectively measured moderate-vigorous physical activity, and total energy intake, and all findings persisted (Supplementary Table S1)

## DISCUSSION

The main findings of the present study are that the capacity to oxidase fat in basal conditions and during exercise (i.e., MFO), are positively associated with plasma S-klotho, while BCHox is inversely associated with the latter, in middle-aged sedentary adults. Neither BMR nor fuel oxidation showed any association with chronological age, either in basal conditions or during exercise.

BMR accounts for ~70% of total energy expenditure, and is largely responsible for overall energy homeostasis [19]. BMR falls by 1-2% per decade after 20 years of age, and is closely linked to the progressive reduction in lean mass seen with ageing [20]. Our group recently reported a strong association between lean mass and plasma S-klotho [21] but, paradoxically, no association was seen between BMR and plasma S-klotho in the present work. This might be explained in that the age of the present subjects was quite homogeneous, and because factors (in addition to the lean mass) such us energy flux rates, mitochondrial proton leakage, protein turnover, and Na+–K+–ATPase activity can influence the BMR during ageing [22]. Future studies with larger sample sizes and with a wide range of subject ages are needed to confirm these findings, and to examine whether changes in BMR are associated with changes in plasma S-klotho.

Metabolic flexibility, defined as the ability to increase fat oxidation upon increased fatty acid availability (decreasing carbohydrate oxidation), and/or to switch between fat and carbohydrate oxidation as the primary fuel source [19], undergoes important changes during the ageing process [20]. Ageing is characterized by a progressive qualitative and quantitative decline in lean mass, poor mitochondrial volume and efficiency, a reduction in type II muscle fibre size, lower capillary density, resistance to anabolic endocrine signals, and a more pro-inflammatory environment [4]. Together, these changes underlie the theoretical framework for the appearance of metabolic inflexibility with ageing, observed through an increased carbohydrate oxidation in different situations. Conflicting results have been reported over time regarding the relationship between basal fuel oxidation and ageing. Initially, some studies reported a reduced BFox in older individuals compared to their younger counterparts [21,22]. However, methodological issues may have influenced these findings (e.g., small sample sizes, narrow and limiting inclusion criteria, poorly defined age groups (young vs. old), different data collection methods, and the method of determining fuel oxidation, etc.). In response, Siervo et al. [3], recently conducted an elegant study to examine the association between BFox and chronological age in a large cohort (3442 individuals (2465 women) aged 18-81 years), using a ventilated-hood indirect calorimetry system to determine fuel oxidation. In agreement with the present findings, but contrary to their own hypothesis, these authors found no significant association between BFox and chronological ageing [3]. They suggested this lack of association might be explained by age-related changes in metabolic flexibility becoming more evident when the fuel oxidation capacity becomes crucial in the regulation of metabolic homeostasis (i.e., in the post-prandial state) [3]. Although ageing has typically been understood in terms of chronological age, several studies have suggested that it is a crude yardstick given the heterogeneity in individuals' physiology and health-related outcomes; further, the influence of ageing is different between individuals, and even at the organ/tissue level of the same individual [10]. Measuring biological ageing biomarkers might therefore provide a more valid and reliable tool for assessing and examining the ageing process [10].

S-klotho is understood to be a powerful anti-ageing biomarker. It functions as a human ageing-suppression molecule and has pleiotropic activities that result in the protection of tissues and organ [23,24] Indeed, previous studies have reported a positive relationship between plasma S-klotho and life span [25], and an inverse association with coronary artery disease [26], atherosclerosis [26], osteoporosis [27], calcinosis, stroke [28], acute and chronic kidney diseases [29], different cancers [30], salt-sensitive hypertension [31] and all-cause mortality [31]. The transmembrane klotho protein is an essential component of endocrine fibroblast growth factor (FGF) receptor complexes, which have a key role in the pathophysiology of ageing-related disorders via the mediation of phosphate and calcium homeostasis [24]. However, S-klotho cannot function as a soluble receptor of FGF, and a number of FGF-independent functions have been described for it in the homeostasis of energy metabolism [14,15,24]. The anti-ageing properties of S-klotho have been thought partially owed to its specific metabolic function: 1) It inhibits insulin and insulin-like growth factor I (IGF-1) receptors, preventing their phosphorylation by the modification of their glycans [12]. Insulin induces transmembrane klotho shedding, and the consequent increase in plasma S-klotho inhibits insulin signalling in peripheral tissues and impedes the prolonged action of insulin [15,24]. This partial inhibition of insulin and IGF-1 is an evolutionarily conserved mechanism for suppressing ageing via the enhancement of insulin sensitivity [15,24]. 2) After binding to different Wnt ligands, it inhibits Wnt signalling and promotes stem cell proliferation and survival [32]. 3) It increases resistance to oxidative stress by inhibiting FOXO phosphorylation and upregulating a number of antioxidant enzymes [33,34].

Recent studies have shown that S-klotho production is downregulated in persons with diabetes mellitus type II; such patients experience hyperglycaemia, insulin resistance and an attenuated resistance to oxidative stress [18]. The reduced presence of S-klotho in these individuals who are metabolically inflexible in response to different stressors [19] hints at metabolic flexibility and plasma S-klotho levels being closely associated - and the present work shows a strong association between metabolic flexibility (both under post-fast baseline conditions and during exercise) with plasma S-klotho.

The present work suffers from a number of limitations. Given its cross-sectional design, no causal interpretation can be established; the sample size in relatively small; and only sedentary adults aged 40-65 years were included. These findings may not be extrapolatable to older, younger, and/or trained individuals.

In summary, the present results suggest that BFox and MFO are positively associated with the plasma S-klotho concentration in middle-aged sedentary adults, whereas a negative association was observed between BCHox and plasma S-klotho concentration. However, no relationship was observed between BFox, BCHox and MFO with chronological age under either set of test conditions. These results have clinical implications, and support the idea that fat oxidation in basal conditions and during exercise are powerful predictors of biological ageing. Further studies are needed to examine whether metabolic flexibility in response to other stressors (e.g., the post-prandial state, or after cold exposure) are associated with plasma S-klotho. A longitudinal intervention aiming to improve fat oxidation should be performed to determine whether plasma S-klotho increases in parallel.

## MATERIALS AND METHODS

### Study design and participants

This cross-sectional study was performed as part of the FIT-AGEING project clinicaltrial.gov: ID: NCT03334357) [35]. Eighty-nine middle-aged, sedentary adults were initially recruited, of whom 15 were excluded from analysis due to problems in data collection or usage; the final number of study subjects was therefore 74 (~52% women) (Supplementary Figure S1). Subjects were recruited through advertisements distributed in the form of leaflets and via social networks and electronic media. The inclusion criteria were: (i) age 40-65 years old, (ii) practicing <20 min of physical activity on <3 days per week (self-reported), (iii) to be taking no drug or long-term medication, (iv) to be a non-smoker, (v) to have no cardiometabolic illness, (vi) to not be pregnant, (vii) and to have experienced no significant weight change (<3 kg) in the past 12 weeks.

All subjects gave their written, informed consent to be included in accordance with the latest revision of the Declaration of Helsinki (2013). The study was approved by the Human Research Ethics Committee of the *Junta de AndalucA-a* [0838-N-2017].

### Procedures

All assessments were made at the Sport and Health University Research Institute (iMUDS, Granada, Spain) during September and October of 2016 and 2017. Subject weight and height were measured using a Seca model 799 scale and stadiometer (Seca, Hamburg, Germany), and the body mass index (BMI) calculated as (*weight [kg]/ height^2^ [m]*). Fat mass, visceral adipose tissue mass and lean mass were determined using a Discovery Wi dual-energy X-ray absorptiometer (Hologic, Inc., Bedford, MA, USA). The fat mass index and the lean mass index were calculated as *(fat mass [kg]/ height^2^ [m])* and *(lean mass [kg]/ height2 [m])* respectively.

Subjects were told to arrive at the laboratory in a motor vehicle, and to avoid any moderate/vigorous physical activity in the previous 24 h/48 h respectively; all were required to confirm that they had met this condition. BMR was determined by indirect calorimetry in a peaceful room at 22-24℃ and 35-45% humidity, at between 8 and 10 a.m. following a 12 h fast, using an Ultima CardiO2 metabolic cart (Medgraphics Corp, MN, USA) and employing a neoprene face-mask with no external ventilation [36]. The evening meal consumed by subjects prior to fasting was standardized: an egg omelette with fried tomato and boiled rice. The Ultima CardiO2 metabolic cart device assessed oxygen consumption (VO_2_) using a galvanic fuel cell, and carbon dioxide production (VCO_2_) via non-dispersive infrared analysis using a breath-by-breath system [37]. Prior to the start of BMR assessment, the subjects reclined on a bed for ~30 min in a comfortable supine position, covered by a sheet [38,39]. Meanwhile, a gas calibration using two standard gas concentrations, and a flow calibration using a 3 L calibration syringe, were performed following the manufacturer's instructions. BMR and basal fuel oxidation were measured over a 30 min period in which the participants were instructed to breath normally, neither talking, fidgeting nor sleeping. The first 5 min of each dataset were discarded. The coefficient of variance (CV) for VO_2_, VCO_2_, the respiratory exchange ratio (RER), and minute ventilation, were calculated for 5 min intervals (i.e., from the 1st to the 5th min, from 2nd to 6th, from 3rd to 7th, etc). In accordance with previous studies [40,41], the 5 min periods that met steady-state gas exchange criteria (i.e., CV<10% in VO_2_, CO_2_, and minute ventilation, and CV<10% in RER) were then selected, and the 5 min period with the lowest CV for VO_2_, VCO_2_, RER, and minute ventilation chosen for further analysis (excluding those subjects with a RER of <0.7 or >1.0). Weir's abbreviated equation [42] was used to estimate the BMR, and Frayn equations [43] were used to estimate basal fat oxidation (BFox) and basal carbohydrate oxidation (BCHox) expressed in g/min. The BMR was also calculated with respect to the lean mass (BMR_LM_). The BFox and BCHox were also expressed as a percentage of the BMR.

Maximal fat oxidation during exercise (MFO), and the intensity of exercise that elicits MFO (Fat_max_), were determined via a walking graded exercise test on a H/P/Cosmos Pulsar treadmill (H/P/Cosmos Sports & Medical GmbH, Nussdorf-Traunstein, Germany). The maximum walking speed was assessed following the methodology used in previous studies [44 ―46]. The walking graded exercise test started with a warm-up at 3.5 km/h and a 0% gradient, and the speed then increased by 1 km/h every 3 min until the maximum walking speed was reached. The gradient was then increased by 2% every 3 min until the RER was >1.0. The subjects wore a Model 7400 face mask (Hans Rudolph Inc, Kansas City, MO, USA) equipped with a prevent™ metabolic flow sensor (Medgraphics Corp, Minnesota, USA) connected to the Ultima CardiO_2_ metabolic cart for measuring gas exchange. Gas and flow calibrations were performed following the manufacturer's instructions. VO_2_ and VCO_2_ data were averaged every 10 s using Breeze Suite software v.8.1.0.54. Fat oxidation was calculated from the respiratory quotients during the last 60 s of each stage in the graded exercise test, using standard indirect calorimetry equations [43]. As previously described, MFO and Fatm_ax_ were estimated via a 3rd polynomial curve with fat oxidation as a function of VO_2_max [3046]. The MFO was also determined with respect to lean mass (MFO_LM_).

Following the modified Balke protocol [47], a maximal graded exercise test was used to determine VO_2_max on another day (interval 3-7 days). Subjects were asked: (i) to fast for 3 to 5 h, but eating a complete meal just before, (ii) to avoid drugs and/or stimulants at least 24 h before the test, and (iii) to refrain from moderate and/or vigorous physical activity for 24 h/48 h before the test respectively. Briefly, the test began at a speed of 3.5 km/h (gradient 0%), increasing until reaching 5.3 km/h. The gradient was then increased by 1% every minute, keeping the treadmill speed constant until subject exhaustion. The heart rate was continuously monitored and recorded every 5 s using a Polar RS800 heart rate monitor (Polar Electro Oy, Kempele, Finland).

Blood samples were obtained from the antecubital vein in the morning just before BMR assessment. Plasma S-klotho was determined in ethylene diamine-acetic acid-treated plasma using a solid-phase sandwich enzyme-linked immunosorbent assay kit (Demeditec, Kiel, Germany). Optical density was assessed at 450 nm ± 2 nm. The intra- and inter-assay CV (3-10% each) was determined using two different doses of pure S-klotho protein.

### Statistical analysis

The normal distribution of all variables was confirmed using the Shapiro-Wilk test, visual histograms, Q-Q plots and box plots. The Student t test for unpaired samples was used to examine differences in the results of male and female subjects. Given the aim of the study, and the lack of any significant interaction between sex (all P>0.05), the appropriateness of fitting models for men and women were combined including sex as a covariable.

Simple linear regression models were first used to examine the association of BMR, BMR_LM_, BFox, BCHox, MFO, MFO_LM_, and Fat_max_ with plasma S-klotho. Multiple linear regression analyses were then conducted to study these associations while controlling for potential confounders: (i) Model 1 was adjusted for age; (ii) Model 2 for sex; and (iii) Model 3 for percentage fat mass. These potential confounders were selected on the basis of theoretical considerations and the results of stepwise regression. Simple linear regression was also performed to examine the association of BMR, BMR_LM_, BFox, BCHox, MFO, MFO_LM_ and Fat_max_ with chronological age. All calculations were made using the Statistical Package for the Social Sciences v.22.0 (IBM Corporation, Chicago, IL, USA). GraphPad Prism 5 software (GraphPad Software, San Diego, CA, USA) was used for graphical plots. Significance was set at P≤0.05

## SUPPLEMENTARY MATERIAL

Supplementary Table S1

Supplementary Figure S1
