# Supplementary material for: Association of basal metabolic rate and fuel oxidation in basal conditions and during exercise, with plasma S-klotho: the FIT-AGEING study
Source: Aging (Albany NY). 2019 Aug 7;11(15):5319–33. doi: 10.18632/aging.102100 (PMC6710061; doi:10.18632/aging.102100)
Supplement: Supplementary Table S1 [file aging-11-102100-s002.pdf]

SUPPLEMENTARY MATERIAL

Supplementary Table S1. Association between energy metabolism outcomes with S-Klotho protein and age adjusted by visceral adipose tissue (Model 1), adjusted by VO_2max (Model 2), adjusted by objectively measured moderate-vigorous physical activity (Model 3), and by total energy intake (Model 4).

	**Plasma S-klotho concentration**			
	Model 1	Model 2	Model 3	Model 4
BMR (kcal/day)	0.126	0.142	0.226	0.140
BMR_{LM} (kcal/ $kg_{leanmass}$/day)	0.634	0.146	0.500	0.836
BFox (g/min)	**<0.001**	**<0.001**	**<0.001**	**<0.001**
BFox (% BMR)	**<0.001**	**<0.001**	**<0.001**	**<0.001**
BCHox (g/min)	**<0.001**	**<0.001**	**<0.001**	**<0.001**
BCHox (% BMR)	**<0.001**	**<0.001**	**<0.001**	**<0.001**
MFO (g/min)	**0.024**	**0.014**	**0.032**	**0.023**
MFO_{LM} (g/$kg_{leanmass}$/min)	0.232	0.409	0.254	0.253
Fat_{max} (% VO_2max)	0.109	0.630	0.161	0.112
	**Chronological age**			
	Model 1	Model 2	Model 3	Model 4
BMR (kcal/day)	0.828	0.287	0.758	0.983
BMR_{LM} (kcal/ $kg_{leanmass}$/day)	0.153	0.206	0.128	0.139
BFox (g/min)	0.459	0.519	0.109	0.244
BFox (% BMR)	0.834	0.806	0.116	0.374
BCHox (g/min)	0.692	0.253	0.066	0.618
BCHox (% BMR)	0.975	0.629	0.110	0.407
MFO (g/min)	0.874	0.343	0.896	0.979
MFO_{LM} (g/$kg_{leanmass}$/min)	0.155	0.168	0.147	0.132
Fat_{max} (% VO_2max)	0.275	0.918	0.051	0.655

P value of multiple-regression analysis. Abbreviations: BMR: Basal Metabolic Rate; BMR_{LM}: Basal Metabolic Rate relative to lean mass; BFox: Basal Fat Oxidation; BCHox: Basal Carbohydrate Oxidation; MFO: Maximal Fat Oxidation; MFO_{LM}: Maximal Fat Oxidation relative to lean mass; Fat_{max}: Intensity of exercise that elicits MFO; VO_2max: Maximum Oxygen Uptake.
