# Supplementary material for: Association of basal metabolic rate and fuel oxidation in basal conditions and during exercise, with plasma S-klotho: the FIT-AGEING study
Source: Aging (Albany NY). 2019 Aug 7;11(15):5319–33. doi: 10.18632/aging.102100 (PMC6710061; doi:10.18632/aging.102100)
Supplement: Supplementary Figure S1 [file aging-11-102100-s001.pdf]

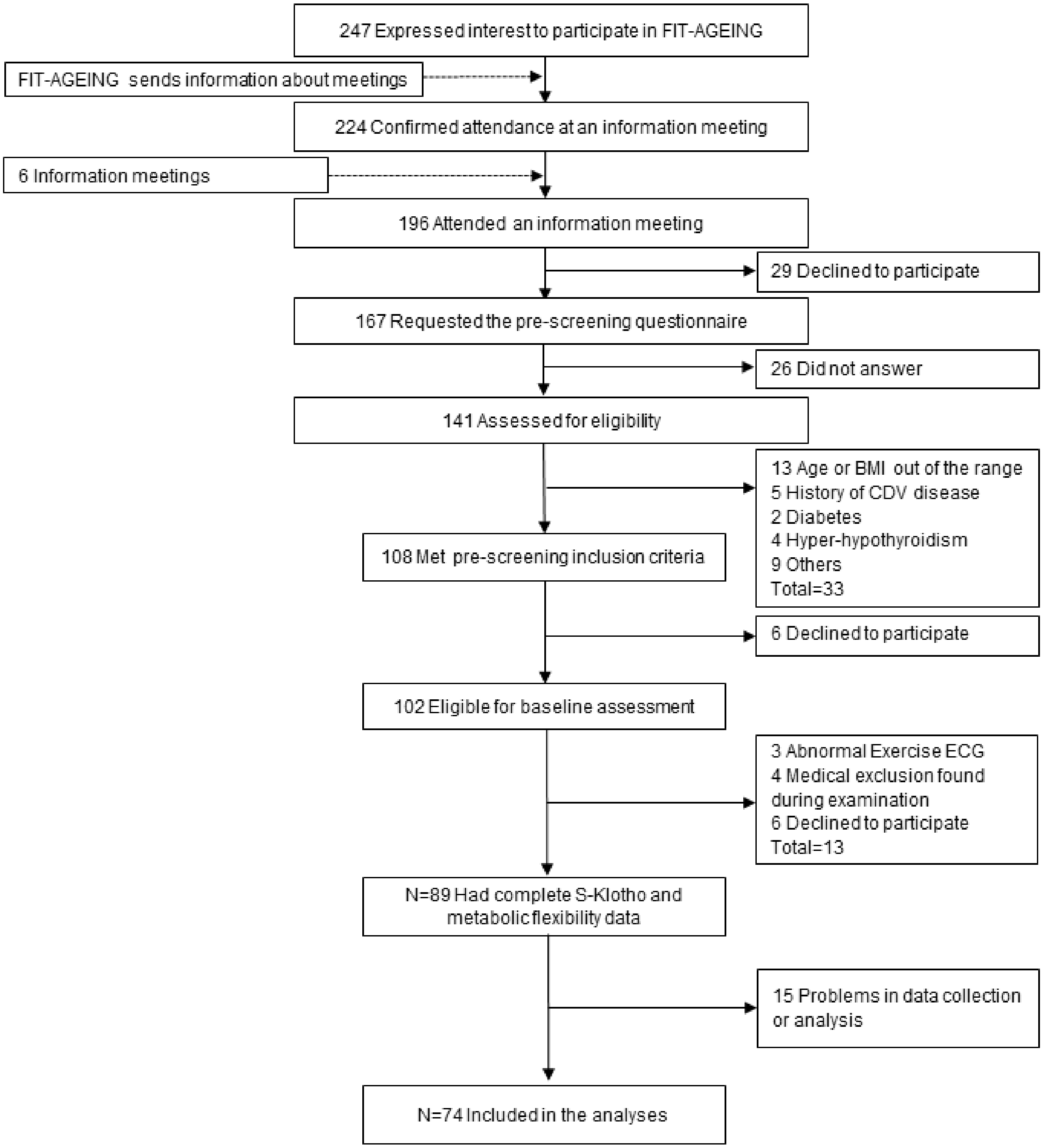

Supplementary Figure S1. Flow-chart showing recruitment of study subjects. BMI: body mass index, CDV: cardiovascular, ECG: electrocardiogram.
